# Beryllium-Ion-Selective PEDOT Solid Contact Electrode Based on 9,10-Dinitrobenzo-9-Crown-3-Ether

**DOI:** 10.3390/s20216375

**Published:** 2020-11-09

**Authors:** Junghwan Kim, Dae Hee Kim, Jin Cheol Yang, Jae Sang Kim, Ji Ha Lee, Sung Ho Jung

**Affiliations:** 1 Central customs Laboratory and Scientific Service, Gyeongnam, Jinju 52851, Korea; ansukido@korea.kr (J.K.); kokanee1@korea.kr (D.H.K.); yang6561@korea.kr (J.C.Y.); 2 Department of Chemistry and Research Institute of Natural Science, Gyeongsang National University, Gyeongnam, Jinju 52828, Korea; jaeskim@gnu.ac.kr; 3 Department of Chemical Engineering, Graduate School of Advanced Science and Engineering, Hiroshima University, 1-4-1 Kagamiyama, Higashi-Hiroshima, Hiroshima 739-8527, Japan; leejiha@hiroshima-u.ac.jp; 4 Department of Liberal Arts, Gyeongnam National University of Science and Technology (GNTECH), Jinju 52725, Korea

**Keywords:** beryllium, crown ether, conducting polymer, all-solid-state electrode, sensor

## Abstract

A beryllium(II)-ion-selective poly(ethylenedioxythiophene) (PEDOT) solid contact electrode comprising 9,10-dinitrobenzo-9-crown-3-ether was successfully developed. The all-solid-state contact electrode, with an oxygen-containing cation-sensing membrane combined with an electropolymerized PEDOT layer, exhibited the best response characteristics. The performance of the constructed electrode was evaluated and optimized using potentiometry, conductance measurements, constant-current chronopotentiometry, and electrochemical impedance spectroscopy (EIS). Under optimized conditions, which were found for an ion-selective membrane (ISM) composition of 3% ionophore, 30% polyvinylchloride (PVC), 64% o-nitro phenyl octyl ether (o-NPOE), and 3% sodium tetraphenylborate (NaTPB), the fabricated electrode exhibited a good performance over a wide concentration range (10^−2.5^–10^−7.0^ M) and a wide pH range of 2.0–9.0, with a Nernstian slope of 29.5 mV/D for the beryllium (II) ion and a detection limit as low as 10^−7.0^ M. The developed electrode shows good selectivity for the beryllium(II) ion over alkali, alkaline earth, transition, and heavy metal ions.

## 1. Introduction

Beryllium (Be) has been widely used in various industries, such as automobile manufacturing, aerospace, nuclear energy, electronics, and communications [1]. Despite its use, Be is extremely hazardous to human health, causing serious diseases including berylliosis and chronic beryllium disease (CBD), which is an incurable and fatal progressive lung ailment [2]. Therefore, it is necessary to develop stable, selective, and sensitive electrochemical techniques methods for the rapid detection of Be [3]. In this regard, although the analytical technique for the ion-selective electrodes has been widely applied to determine the chemical analytes, research on beryllium ion-selective electrodes is sparse [4,5,6]. This is due to the tendency of hydration causing a small size of beryllium ion in aqueous solution. Therefore, designing an ion-selective membrane (ISM) suitable for beryllium ions remains a challenging task.

Along these lines, we focus on all-solid-state ion-selective electrodes (ASS-ISEs) [3,7,8], which are drawing attention as alternatives to conventional electrodes with an inner solution. In ASS-ISEs, ion-to-electron transduction takes place in a solid and compact layer of conductive polymer, which is placed into contact between the ion-selective membrane (ISM) and the electron-conducting metallic substrate. ASS-ISEs can be placed in positions that pose no risk of leakage of the inner solution. Moreover, the fabrication procedures involving ASS-ISEs are much simpler than those of conventional solid-contact ISEs, and no additional steps are required to produce the intermediate layer [9].

Coated-wire electrodes (CWEs) [10] were initially used in solid-contact ISEs, but such ISEs exhibited a poor potential stability due to the blocked interface between the electron conductor and the ion-selective membrane [11]. Therefore, various electroactive materials have been proposed for this interface to improve the potential stability [12]. Several solid-contact transducers have been introduced, including hydrogels [13], redox-active self-assembled layers [14], carbonaceous materials [15], fullerene [16], graphene [17], as have conducting polymers, such as polypyrrole [18], polyaniline [19,20,21], polythiophene [22,23], and poly(3-methylthiophene) [24,25,26,27].

Among these materials, the conductive polymer PEDOT is one of the most promising ion-to-electron transducers for solid-contact ISEs, since PEDOT offers less electroactivity and fewer electrochemical side reactions than the highly *p*-doped conductive polymers [28,29]. Moreover, PEDOT is highly conductive, which may assist in preventing the accumulation of water and salt between the conductive substrate and the ISM.

In this work, we report an ASS-ISE based on PEDOT, with a benzo crown ether as a neutral carrier, that exhibits high beryllium selectivity and sensitivity. Employing PEDOT as an active ion-to-electron transducer in combination with an ISM forms an excellent strategy for beryllium sensing while avoiding the detrimental water layer formed at the buried interface of ASS-ISEs.

## 2. Materials and Methods

### 2.1. Reagents and Preparation of Solution

Beryllium sulfate tetrahydrate was purchased from Alfa Aesar. The membrane components, monomeric 3,4-ethylenedioxythiophene (EDOT), high-molecular-weight poly(vinyl chloride) (PVC), o-nitro phenyl octyl ether (o-NPOE), and sodium tetraphenylborate (NaTPB), were obtained from Aldrich. All other chemicals were of analytical grade and used without further purification. All beryllium solutions were prepared from distilled-deionized water with a resistance of ≥18.2 MΩ, and solutions of different concentrations were prepared using the sulfate salts of given cations with diluting 0.1 M stock solutions.

### 2.2. Instrumentation 

Nuclear magnetic resonance (NMR) spectra were recorded on a Bruker Avance-300 (300 MHz) spectrometer. High-resolution mass spectra were measured using a JEOL JMS-700 (MStation) instrument. Conductance measurements were performed using a Metrohm 660 conductivity meter at a frequency of 2 kHz, with the cell constant of 0.769 cm^−1^ was used. The reference electrode (Orion sleeve-type double-junction Ag/AgCl reference electrode; model 90-02) was used to compare with the ISEs; the potential differences were conducted using a PC equipped with a high-impedance-input 16-channel analog-to-digital converter (KOSENTECH, Korea). Electrochemical experiments were carried out at room temperature using a PARSTAT2263 (Princeton Applied Research, Oak Ridge, TN, USA) with a three-electrode system. A saturated Ag/AgCl electrode was used as the reference, and all potentials were recorded with respect to this electrode.

### 2.3. Synthesis of the Ionophore 

Compound **1** (benzo-9-crown-3-ether) was synthesized according to previously reported procedures [29]. Briefly, benzo-9-crown-3-ether (2.0 g, 1.1 mmol) in CH_2_Cl_2_ (50 mL) was added to glacial acetic acid (30 mL), and the mixture was stirred with one drop of 96% H_2_SO_4_. Afterward, 70% HNO (10 mL) was slowly added to the mixture, followed by stirring at RT for 1 h and refluxed for 4 h. After cooling to 0 °C in an ice bath, aqueous Na_2_CO_3_ was added to adjust the pH to 7. Following extraction with CHCl_3_, the organic solvent was evaporated, and the remaining extract was washed with a saturated NaHCO_3_ solution. The crude product was recrystallized from absolute ethanol, affording a pure product with a 59% yield after filtration and drying. MP: 94.5 °C; 1H-NMR (300 MHz), DMSO: δ 7.7 (s, 2H, ArH), 4.4 (m, 4H), 3.85 (m, 4H); MS: m/z (%) 270.20 (M+, 100).

### 2.4. Conductance Measurements

BeSO_4_ solution (5.0 × 10^−5^ M) was placed in the conductometric cell in a mixed solvent (7 mL) of 95% Acetonitrile-DMSO and titrated with a solution of **1** in the same solvent to a molar ratio of [**1**]/[Be^2+^] ranging from 5.0 × 10^−4^ M to 3 of mole ratio. Conductance readings were recorded at 25 °C. Similar procedures were followed for other metal cations, M^n+^, to obtain plots of molar conductance versus [**1**]/[M^n+^] concentration ratio. The stability constants (log *K_f_*) were calculated by fitting all the conductometric curves with a nonlinear least-squares curve fitting program, KINFIT [30].

### 2.5. Electrode Preparation and ISE Measurements

The polymerization of Poly(3,4-ethylenedioxythiophene) (PEDOT) was carried out galvanostatically in acetonitrile solutions containing the 20 mM of EDOT monomer in the presence of 100 mM of TBAP (tetrabutylammonium perchlorate), while EDOT was polymerized electrochemically onto Pt substrates using the cyclic voltammetry (CV) technique and following reported procedures [8]. 

To prepare all-solid-state ISEs, a solution of **1** (100 μL) was applied to bare Pt and Pt/PEDOT electrodes. The membrane was composed of ~3.0 mg of **1**, ~3.0 mg of NaTPB, ~64.0 mg of o-NPOE, and ~30.0 mg of PVC. These components were dissolved in 1.5 mL of THF. After the evaporation of THF, the resulting plasticized PVC membrane covered the underlying PEDOT film, yielding Pt/PEDOT/1-ISM (**E1**) and Pt/1-ISM (**E2**) electrodes. 

For electromotive force (EMF) measurements, the dynamic response curves and calibration plots of ISEs were obtained, and a standard solution was added stepwise to 100 mL of background electrolyte under stirring at 25 °C. The calibration curve for Be^2+^ is shown in Figure S1.

The selectivity coefficients towards several cations were determined by a separate solution method (SSM) using the reduced form of the Nikolsky–Eisenman equation [31].

$$\log K_{i,j}^{pot}=\frac{\left(E_{j}-E_{i}\right)}{S_{i}}$$

Here, *E_j_* and *E_i_* are the potentials measured in a 10−2.5 M solution of the interfering ion and a 10−2.5 M solution of the primary ion (M^n+^), respectively. *S_i_* is the calibration slope. The detection limits of the electrodes were estimated through a method suggested by IUPAC [32].

### 2.6. Chronopotentiometry Measurement

For constant-current chronopotentiometric measurements, the experiments were performed using cyclic voltammetry at 25 °C. To investigate the **E1** and **E2** electrodes in 0.1 M of BeSO_4_ solution as a function of time, a constant current of +1.0 nA for 60 s followed by −1.0 nA for another 60 s was applied while the potential of electrodes was measured. 

### 2.7. Impedance Measurements

Electrochemical impedance spectroscopy (EIS) measurements were performed in deaerated 0.1 M BeSO_4_ solution with the same voltammetric cell and electrodes under quiescent conditions. The impedance spectra were recorded in the frequency range of 100 kHz to 10 mHz using a sinusoidal excitation signal with an amplitude of 10 mV. 

## 3. Results and Discussion

### 3.1. Binding Studies

**Figure 1:**
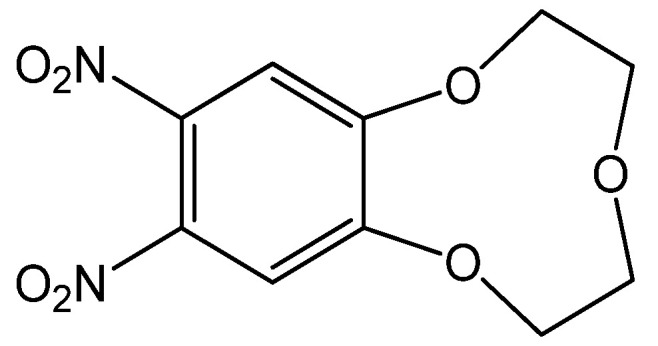
Chemical structure of **1**.

**Figure 2:**
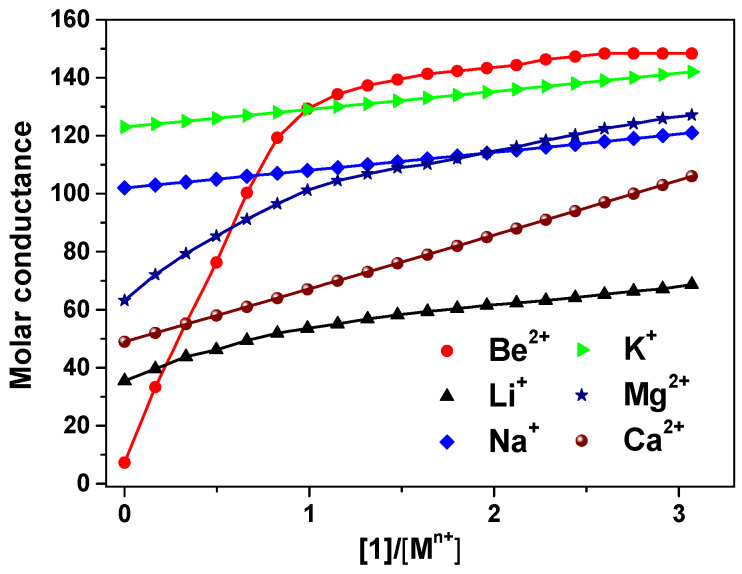
Conductometric titration curves for **1** with metal cations, obtained in 95% acetonitrile-DMSO (AN-DMSO) solution. The molar conductance Λ_m_ (S^−1^cm^2^mol^−1^) is plotted against [**1**]/[M^n+^].

The 9-crown-3-ether moiety of ionophore **1** is widely known as a receptor for the Be^2+^ ion due to its cavity size (0.26 Å) (Figure 1) [5,33,34]. In order to investigate the binding ability between the Be^2+^ ion and **1**, we performed conductance measurements that determined the stoichiometry and stability constants for the complexes formed between **1** and Be^2+^ salts. Figure 2 shows the plots of molar conductance, Λ_m_, versus the metal cation concentration ratio [**1**]/[M^n+^], where M^n+^ was Be^2+^ and other cations. As shown in Figure 2, the Be^2+^ binding curve for **1** obviously plateaued starting at a 1:1 mole ratio. The stability constants of the complex between **1** and the metal cations can be determined by analyzing the molar conductance data using the KINFIT program. A best-fit curve for **1**-Be^2+^ was obtained by a nonlinear least-squares fitting procedure. As shown in Table 1, the stability constant between **1** and the Be^2+^ ion (log *K_f_* = 6.54) was higher than that of the other metal cations studied, indicating that **1** a exhibited greater selectivity for Be^2+^ than for other metal cations. The order of the stability constants is as follows: Be^2+^ >> Mg^2+^ > Li^+^ > Ca^2+^ > Na^+^ > K^+^. These results indicate that ionophore **1** can be employed in electrodes as an ion-selective membrane for the Be^2+^ ion.

### 3.2. Response and Selectivity of All-Solid-State Be^2+^-ISE

To optimize several parameters related to the performance of the ASS-ISEs, ionophore **1** was mixed and tested with different plasticizers: *o*-NPOE, NPPE, TEHP, and DBP. The best membrane performance was obtained in compositions with 3% ionophore, 3% NaTPB, 64% *o*-NPOE, and 30% PVC. The performance of the prepared ISEs was analyzed in metal cation solutions with concentrations ranging from 10^−2.0^ to 10^−8.0^ M.

**Figure 3:**
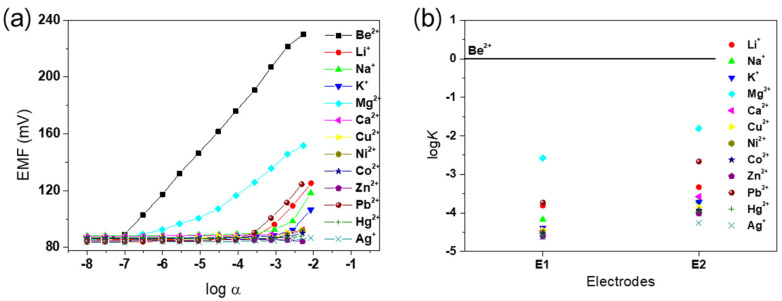
(**a**) Calibration curve of the fabricated Be^2+^ ion-selective electrode (ISE), consisting of the potentiometric response of **E1** for various metal cations. (**b**) Potentiometric selectivity coefficients ($\log K_{Be^{2+},M^{n+}}^{pot}$) for **E1** and **E2**.

To confirm the desired effect of PEDOT on the membrane as an ion-to-electron transducer, two different electrodes, **E1** (with PEDOT) and **E2** (without PEDOT), were investigated in this study. In potential measurements, a linear response with a near-Nernstian slope of 29.5 mV/decade was observed between 10^−2.5^ M and 10^−7^ M Be^2+^ for **E1**, while **E2** gave a sub-Nernstian response (data not shown). The results obtained for alkali, alkaline-earth, and transition metal cations are shown in Figure 3a. The slopes, linear ranges, detection limits, and response times of **E1** and **E2** are shown in Table S1. The Be^2+^ detection limit of **E1**, calculated from the intersection of the two slopes, was 10^−7.0^ M, which is superior to the detection limit of **E2** of 10^−6.2^ M. In addition, **E1** exhibited a faster response time than **E2** (Figure S2). Thus, electrode **E1** clearly demonstrated improved selectivity and sensitivity for the Be^2+^ ion, consistent with the presence of ionophore **1**. As shown in Figure 3b, the potentiometric selectivity coefficients of the **E1** and **E2** electrodes towards several metal cations were determined by the separate solution method (SSM). Electrode **E1** showed significantly enhanced selectivity for Be^2+^ ($\log K_{Be^{2+},M^{n+}}^{pot}$ > −3.7) over other metal cations compared with electrode **E2**. These results bear out the rational prediction of improved electrochemical performance through efficient ion-to-electron transduction via PEDOT on the membrane.

To clarify the significant role of PEDOT, the potential stability of the all-solid-state Be^2+^-ISE was studied using the constant-current chronopotentiometric measurement suggested by Bobacka [11]. Chronopotentiograms were recorded for **E1** and **E2** (Figure S3). The potential drift of electrode **E1**, derived from the ratio Δ*E*/Δ*t*, was 1.62 μV/s at a current of 1 nA, which was lower than that of **E2**, for which Δ*E*/Δ*t* = 4.18 μV/s at the same current. This result suggests that potential stability can be dramatically improved by applying PEDOT as the solid contact between the Pt electrode and the ion-selective membrane. Additionally, the **E1** and **E2** electrodes were characterized by electrochemical impedance spectroscopy (EIS). As shown in Figure S4b, the introduction of the conductive PEDOT between the Pt electrode and the ion-selective membrane reduced the resistance from 12.2 MΩ to 9.3 MΩ, which is evidence that PEDOT facilitates charge transport across the interfaces of ASS-ISMs—that is, it “unblocks” the interface.

### 3.3. pH Effects

**Figure 4:**
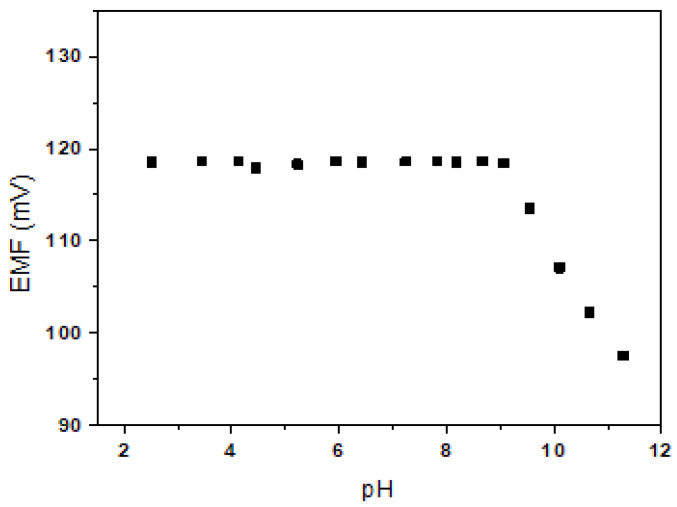
Effect of pH on **E1**.

The effect of pH on the Be^2+^-ISE (**E1**) was tested in a 10^−2^ M BeSO_4_ solution over the pH range of 2.5–11 (adjusted with HNO_3_ or NaOH). As shown in Figure 4, the potential response was almost the same over the pH range of 2.5–9.0. The observed drift at higher pH values may be due to the formation of hydroxyl complexes with Be^2+^ ions in solution. Thus, the wide pH working ranges of the developed electrode **E1** may be useful for selective Be^2+^ sensing in aqueous solution.

### 3.4. Analytical Applications 

Electrode **E1** was used as an indicator electrode in a titration of 10^−4^ M BeSO_4_ with a standard 10^−2^ M EDTA solution, allowing the accurate determination of the amount of Be^2+^ ion in solution, as evidenced in the titration curve in Figure S5. In addition, **E1** was maintained in a 0.01 M Be^2+^ solution for 2 months to investigate its long-term response behavior, and there were no drastic changes in the slope or the detection limit of the electrode (Figure S6). Thus, the proposed Be^2+^-ISE exhibited a good performance for at least 1 month under laboratory conditions.

## 4. Conclusions

This study shows that PEDOT can serve as an effective ion-to-electron transducer in all-solid-state Be^2+^-ISEs in combination with a plasticized PVC-based membrane containing ionophore **1.** The electrochemical properties of an all-solid-state electrode (**E1**) were quantitatively improved by the introduction of PEDOT, as shown by comparison with an electrode (**E2**) where PEDOT was absent. The proposed electrode exhibited stable Nernstian characteristics in BeSO_4_ solutions with concentrations ranging from 10^−2.5^ to 10^−7^ M, and response times shorter than 15 s. Therefore, the developed electrode with a wide working pH range from 2.5 to 9.0, based on incorporating PEDOT into an ion-selective membrane containing ionophore **1**, is well suited for applications requiring highly selective beryllium sensors.

## Figures and Tables

**Table 1:** Log *K_f_* values of binary mixtures of **1** complexes with different cations in 95% AN-DMSO.

	Li^+^	Na^+^	K^+^	Be^2+^	Mg^2+^	Ca^2+^
**1**	4.05	3.05	2.88	6.54	4.51	3.45

## Supplementary Materials

The following are available online at https://www.mdpi.com/1424-8220/20/21/6375/s1: Figure S1: Calibration curve obtained by increasing Be^2+^ ion for the **E1**; Figure S2: Dynamic response time of (**a**) electrode **E1** and (**b**) **E2** for step changes in concentration of Be^2+^ ion; Figure S3: Chronopotentiograms for the **E1** (black line) and the **E2** (red line) electrodes recorded in 10^−3^ M BeSO_4_. The applied current was +1 nA for 60 s and −1 nA for 60 s.; The total resistance (*R*) of the **E1** electrode is approximately 9.5 MΩ, estimated by the potential jump, according to Ohm’s law, *R* = *E*/*I*, where *E* represents the potential change and *I* is the applied current; Figure S4: Impedance spectra of (a) the bared Pt (solid circle) and the PEDOT/Pt (hollow circle) recorded in 0.1 M KCl, the frequency range, 10 mHz–100 kHz; the excitation amplitude, 10 mV; The sharp of the impedance spectra is typical for PEDOT film in an aqueous electrolyte. The impedance spectra are dominated by an approximate 90° capacitive line, and there is only a slight deviation from the capacitive line at high frequencies. These results indicate that a fast electronic transfer occurs at the interface between Pt/PEDOT and PEDOT/solution. Also, the redox capacitance was estimated using the equation C_LF_ = 1/(2π*f*Z″), where *f* is the lowest frequency used to record the spectra (10 mHz), and Z″ is the imaginary part of the impedance at this frequency. The calculated C_LF_ was 617 and 239 μF for Pt/PEDOT and bared Pt, respectively. And (b) the **E1** (solid circle) and **E2** (hollow circle) and the electrodes recorded in 10^−3^ M BeSO_4_ at the open-circuit potential. The frequency range, 10 mHz–100 kHz; the excitation amplitude, 100 mV; Figure S5: Potential titration curve of the **E1** as an indicator electrode, condition: 25 mL of 0.1 mM BeSO_4_ with 10 mM EDTA; Table S1: Response characteristics of all-solid state electrode (**E1**) and coated wire electrode (**E2**); Figure S6: Long-term response behavior of the electrode **E1**.
