# Supplementary material for: Beryllium-Ion-Selective PEDOT Solid Contact Electrode Based on 9,10-Dinitrobenzo-9-Crown-3-Ether
Source: Sensors (Basel). 2020 Nov 9;20(21):6375. doi: 10.3390/s20216375 (PMC7664937; doi:10.3390/s20216375)
Supplement: Supplementary file 1 [file sensors-20-06375-s001.pdf]

Supporting Information

Beryllium-Ion-Selective PEDOT Solid Contact Electrode Based on 9,10-Dinitrobenzo-9-Crown-3-Ether

Junghwan Kim [1], Dae Hee Kim [1], Jin Cheol Yang [1], Jae Sang Kim [2], Ji Ha Lee [3] and Sung Ho Jung [4,*]

[1] Central customs Laboratory and Scientific Service, Gyeongnam, Jinju 52851, Korea; ansukido@korea.kr (J.K.); kokanee1@korea.kr (D.H.K.); yang6561@korea.kr (J.C.Y.)
[2] Department of Chemistry and Research Institute of Natural Science, Gyeongsang National University, Gyeongnam, Jinju 52828, Korea; jaeskim@gnu.ac.kr
[3] Department of Chemical Engineering, Graduate School of Advanced Science and Engineering, Hiroshima University, 1-4-1 Kagamiyama, Higashi-Hiroshima, Hiroshima 739-8527, Japan; leejiha@hiroshima-u.ac.jp
[4] Department of Liberal Arts, Gyeongnam National University of Science and Technology (GNTECH), Jinju 52725, Korea
* Correspondence: sungho@gntech.ac.kr

Received: 15 October 2020; Accepted: 6 November 2020; Published: date

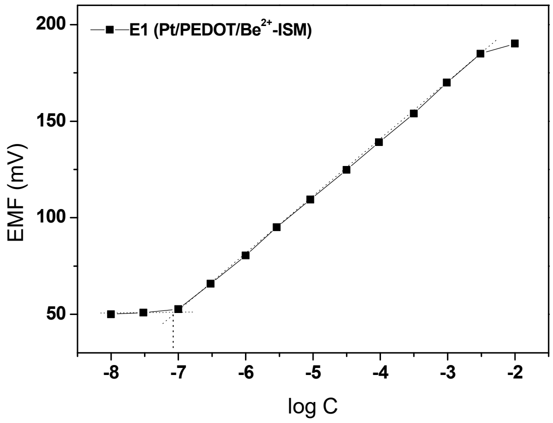

Figure S1. Calibration curve obtained by the increasing Be^{2+} ions for the **E1**.

Table S1. Response characteristics of all-solid-state electrode (**E1**) and electrode (**E2**)

Electrode	Ionophore (%)	PVC (%)	*o*-NPOE (%)	NaTPB (%)	Slope (mV/D)	Detection limit (M)	Linear range (M)	Response time (s)
**E1**	3	30	64	3	29.5	$10^{-7.1}$	$10^{-2.5}$-10^{-7}	15
**E2**	3	30	64	3	25.3	$10^{-6.2}$	$10^{-2.5}$-10^{-6}	35

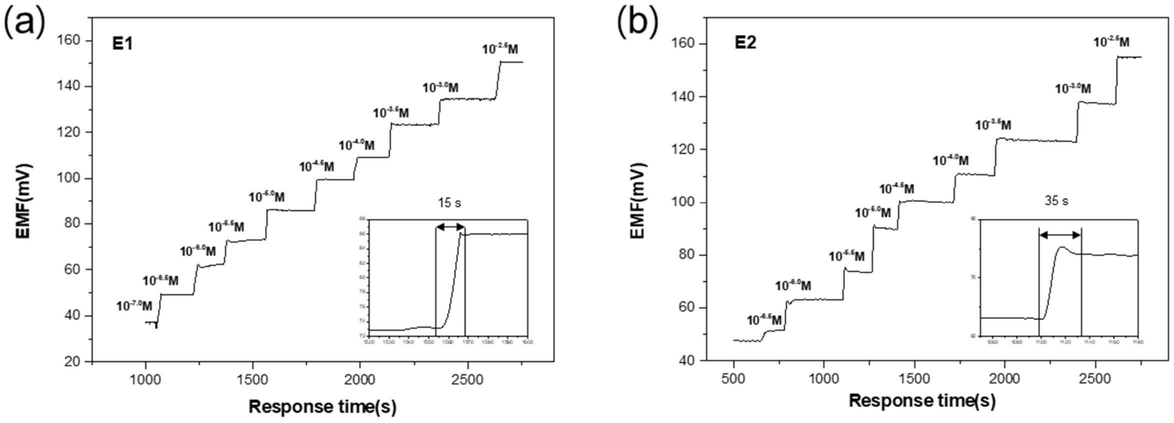

Figure S2. Dynamic response time of (**a**) electrode **E1** and (**b**) **E2** for step changes in concentration of Be^{2+} ion.

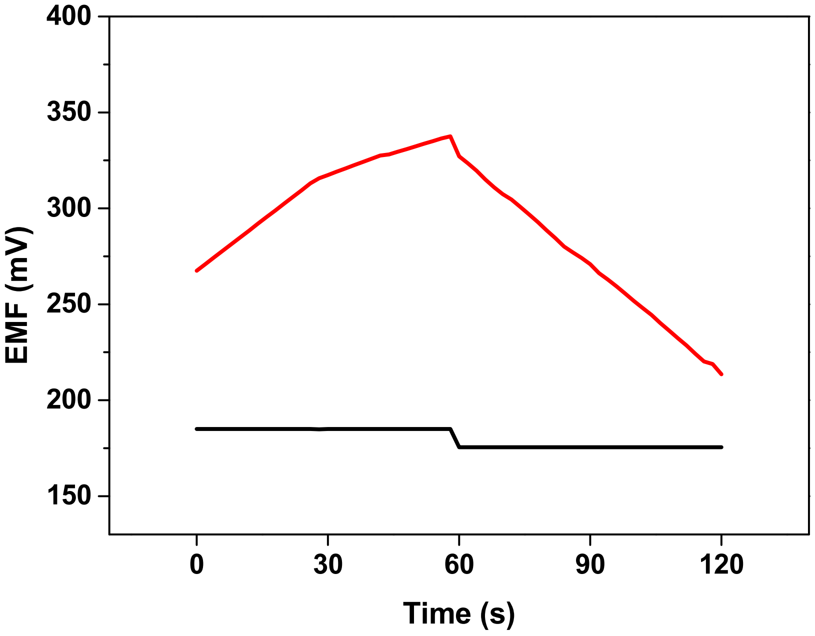

Figure S3. Chronopotentiograms for the **E1** (black line) and the **E2** (red line) electrodes recorded in 10^{-3} M of $BeSO_4$. The applied current was +1 nA for 60 s and −1 nA for 60 s. The total resistance (R) of the **E1** electrode is approximately 9.5 MΩ, estimated by the potential jump, according to Ohm's law, R=E/I, where E represents the potential change and I is the applied current.

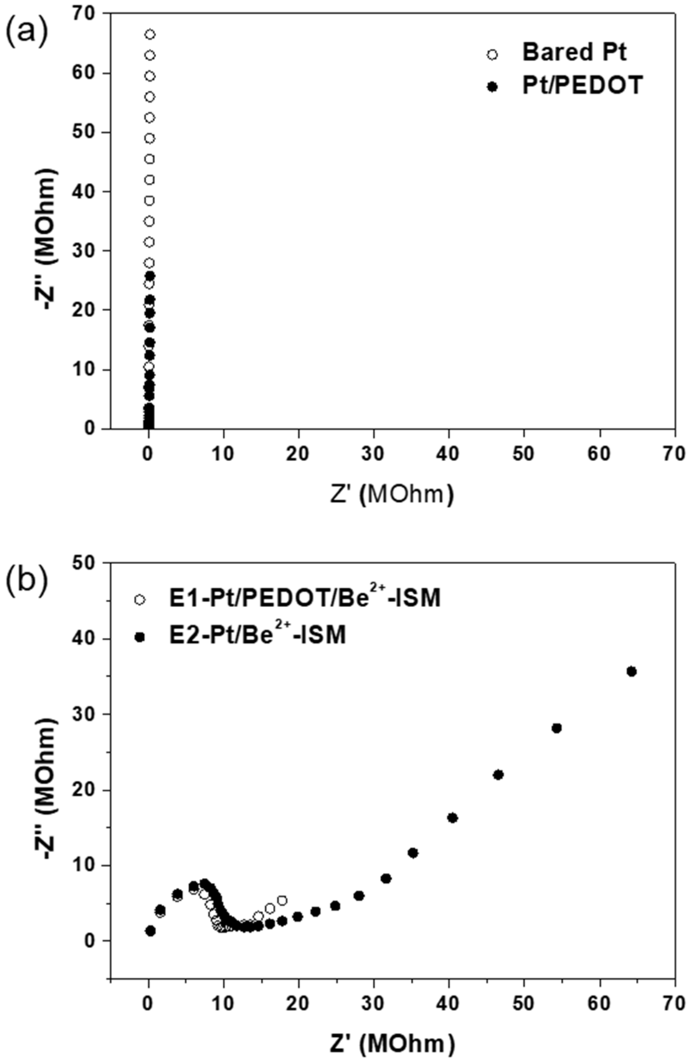

Figure S4. Impedance spectra of (a) the bared Pt (solid circle) and the PEDOT/Pt (hollow circle) recorded in 0.1 M of KCl, with the frequency range 10mHz to 100kHz and the excitation amplitude, 10 mV. The sharp impedance spectra is typical for PEDOT film in an aqueous electrolyte [1]. The impedance spectra are dominated by an approximate 90º capacitive line, and there is only a slight deviation from the capacitive line at high frequencies. These results indicate that a fast electronic transfer occurs at the interface between Pt/PEDOT and PEDOT/solution. Additionally, the redox capacitance was estimated using the equation $C_{LF}=1/(2\pi f Z'')$, where f is the lowest frequency used to record the spectra (10mHz), and Z" is the imaginary part of the impedance at this frequency. The calculated C_{LF} was 617 and 239 µF for Pt/PEDOT and bared Pt, respectively, and (b) the **E1** (solid circle) and **E2** (hollow circle) and the electrodes recorded in 10^{-3} M $BeSO_4$ at the open-circuit potential with the frequency range 10mHz to 100kHz and the excitation amplitude, 100 mV.

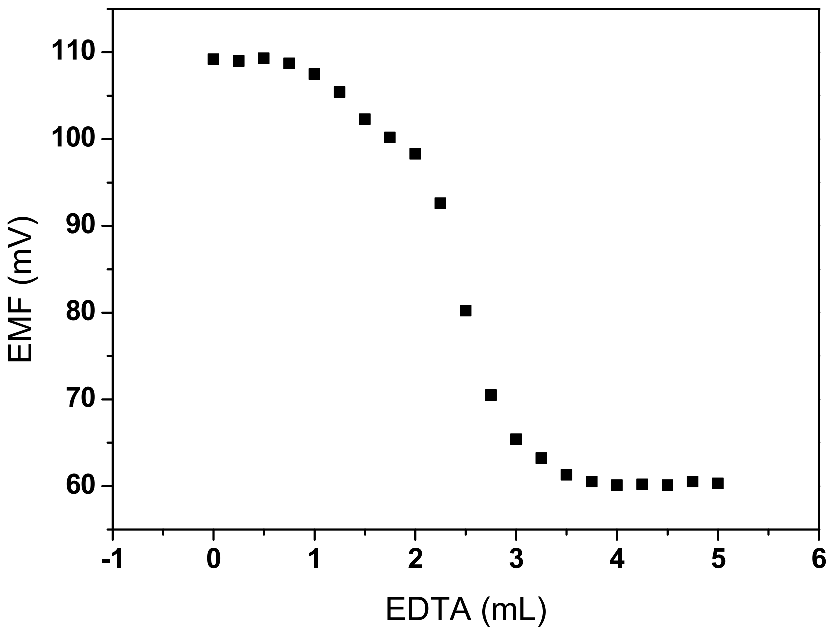

Figure S5. Potential titration curve of the **E1** as an indicator electrode, condition: 25 mL of 0.1 mM $BeSO_4$ with 10 mM of EDTA.

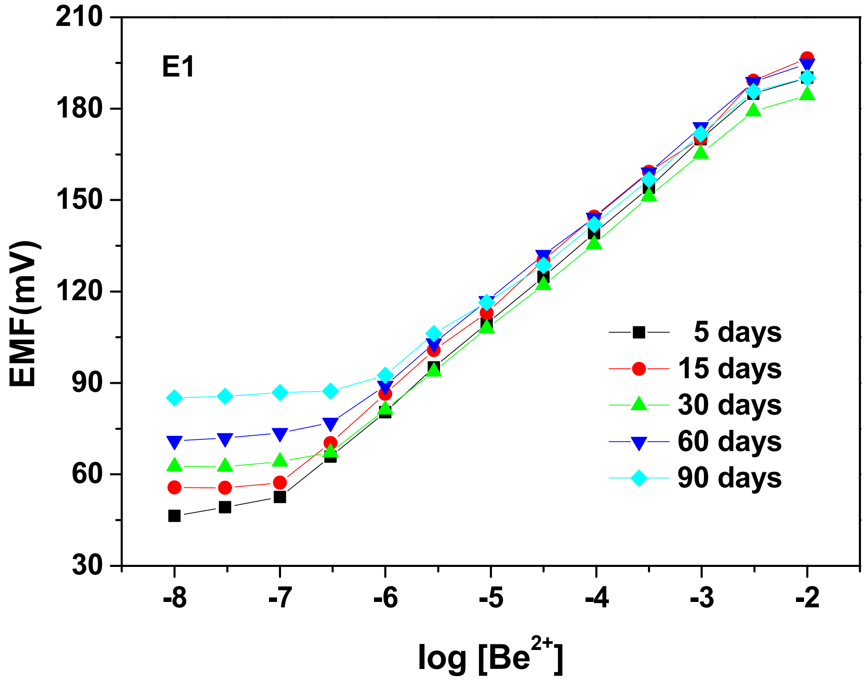

Figure S6. Long-term response behavior of the electrode **E1**.

References

1. Jasielec, J. J.; Sokalski, T.; Filipek, R.; Lewenstam, A. Comparison of different approaches to the description of the detection limit of ion-selective electrodes. *Electrochimica. Acta*. **2010**, *55*, 6836.
